# Factors associated with Burnout Syndrome in police officers: a scoping review

**DOI:** 10.1590/0034-7167-2023-0444

**Published:** 2024-07-15

**Authors:** Beatriz Maria dos Santos Santiago Ribeiro, Fabio Scorsolini-Comin, Maria Lucia do Carmo Cruz Robazzi, Sérgio Valverde Marques dos Santos, Fábio de Souza Terra, Rita de Cassia de Marchi Barcellos Dalri

**Affiliations:** I Universidade de São Paulo. Ribeirão Preto, São Paulo, Brazil; II Universidade do Estado de Minas Gerais. Passos, Minas Gerais, Brazil; III Universidade Federal de Alfenas. Alfenas, Minas Gerais, Brazil

**Keywords:** Public Security, Psychological Exhaustion, Cops, Worker’s Health, Work, Seguridad Pública, Agotamiento Psicológico, Policías, Salud Ocupacional, Enfermería

## Abstract

**Objectives:**

to synthesize research on factors associated with Burnout Syndrome (BS) in police officers.

**Methods:**

a scoping review was conducted without temporal or language restrictions. Data were exported to EndNote to remove duplicates and then imported into the Rayyan app for organization, article selection, and data extraction.

**Results:**

a total of 4559 publications were identified, with 50 studies included in the review. Research conducted in Brazil and the United States predominated. Certain occupational factors were found to be more closely linked to police officers compared to other professions, including law enforcement, frequency of interaction with suspects and criminals, rank, dissatisfaction with the organization, and civilian confrontations.

**Conclusions:**

certain aspects of the police profession contribute to BS, even in countries with better working conditions in public security. It is recommended to prioritize health promotion initiatives for these professionals.

## RESUMEN

**Objetivos:**

sintetizar los estudios que abordan los factores asociados al síndrome de burnout (SB) en policías.

**Métodos:**

revisión de alcance, sin restricción temporal y de idiomas con exportación a EndNote, eliminación de duplicados y exportación a la aplicación Rayyan para organización, selección de artículos y extracción de datos.

**Resultados:**

se identificaron 4559 publicaciones con inclusión de 50 estudios. Hubo predominio de investigaciones realizadas en Brasil y Estados Unidos. Algunos factores laborales estuvieron más relacionados con los policías en comparación con otras profesiones, como la aplicación de la ley, la frecuencia de interacción con sospechosos y criminales, ser cabo, insatisfacción con la corporación y enfrentamientos con civiles.

**Conclusiones:**

ciertos aspectos laborales contribuyen al SB, incluso en países con mejores condiciones laborales en Seguridad Pública. Se recomienda priorizar acciones de promoción de la salud para estos profesionales.

## INTRODUCTION

Burnout Syndrome (BS) is caused by physical and mental exhaustion, which can result in negative behaviors^(1)^. The first dimension of BS is emotional exhaustion, marked by a lack of energy, enthusiasm, and a feeling of being drained of solutions. A sense of frustration can arise, with workers feeling exhausted in dealing with clients, perceiving that their performance is not as competent as before^(1)^.

The second dimension is depersonalization, where the professional treats people and the organization as objects, potentially developing emotional insensitivity. The third dimension is low professional achievement, characterized by a tendency for the worker to self-evaluate negatively, feeling unhappy with themselves and dissatisfied with their professional performance, leading to a decline in feelings of competence and success, as well as a decrease in the ability to interact with others^(1)^. Investigating BS allows for the implementation of the concept of integrality of actions in Occupational Health.

In the daily work routine, simultaneous exposure to risk factors for the development of BS is very common. As it affects the worker’s health, leave may be granted, but associated illness should be included as a secondary diagnosis. Recognition of BS in the workplace should be based on the Occupational Accident Report. This syndrome was classified, in 2022, in the International Classification of Diseases (ICD 11) as QD85, in the chapter entitled Problems Associated with Employment and Unemployment; it is outside the chapter referring to mental, behavioral, or neurodevelopmental disorders present in ICD 10^(2)^.

The work of police officers is often risky and unhealthy. In their professional performance, there are factors inherent to the development of BS. Most of the time, they cannot express feelings such as pleasure, restlessness, and fear, for fear of being seen as weak and cowardly. Newly graduated officers are subject to evaluation by a group of superiors, a situation that can be a source of anxiety, pressure, and suffering^(3)^, potentially causing harm to their mental health. The police ensure law enforcement by preserving public order, meeting the security needs of the community, and assisting preventively^(4)^. As a defense mechanism, some police officers adopt a rigid posture not only within the institution in which they work but also in social relationships^(3)^. However, this work requires that these professionals have resilience and good adaptability to deal with and overcome the possible effects of their daily lives, but this is not always the case with all police officers^(5)^.

Police officers are exposed to danger in their routine, when their own lives are at risk, and this tension also extends to their days off. Studies conducted with Brazilian police officers have shown stress and lack of recognition for the effort made^(6-7)^, feelings of frustration, uselessness, and unproductiveness, inadequate working conditions represented by the lack of equipment, shortage of physical facilities, and insufficient human resources^(8)^, influencing the physical and mental health deterioration of these professionals^(9)^.

Given the above, the need to research BS in the field of Public Security work, especially in police officers, was observed, as it is a profession marked by imminent risks and stress, emphasizing the factors related to the causes and symptoms that can lead them to BS. This study can contribute to the health and safety of these workers, reflecting in better security conditions for the population, as well as in improving the quality of life at work for these professionals. It is also hoped that this review can contribute to the development of preventive measures and health promotion actions aimed at this audience.

The research question that guided this study was: what scientific evidence addresses police officers affected by BS?

## OBJECTIVES

To synthesize studies addressing the factors associated with burnout syndrome (BS) in police officers.

## METHODS

### Study design and period

This is a scoping review aimed at mapping the main definitions and scientific evidence available on a specific area/theme^(10)^. The searches were conducted in December 2022.

### Inclusion criteria

The inclusion criteria adopted were: studies written in any language, with no temporal restrictions, and that answered the research question. Studies with different levels of evidence were included, allowing for a more comprehensive understanding of the state of the art on a given issue^(11)^. In order to ensure the integrity of this study and its methodological rigor, the five criteria advocated in the Joanna Briggs Institute (JBI) protocol were followed: establishment of the research question, identification of studies, selection and inclusion of studies, organization of the selected studies, and finally, interpretation, analysis, and compilation of the results^(10)^.

### Study protocol

The existence of similar scoping review protocols on research platforms such as the International Prospective Register of Systematic Reviews (PROSPERO)^(12)^, Open Science Framework (OSF)^(13)^, The Cochrane Library^(14)^, JBI Clinical Online Network of Evidence for Care and Therapeutics (COnNECT+)^(15)^, and Database of Abstracts of Reviews of Effects (DARE)^(16)^ was verified. The results showed the absence of reviews with objectives and contents similar to those of this study.

The PCC strategy (acronym for P: Population = Police officers, C: Concept = Burnout Syndrome, C: Context = Police officers affected by burnout syndrome) was used to develop the guiding question of the study, namely, what scientific evidence addresses police officers affected by BS?

The Rayyan software, recommended for systematic reviews and meta-analyses^(17)^, was used for managing records. Searches were conducted in the Medline/PubMed, EMBASE, Scopus, Web of Science, PsycINFO, Latin American and Caribbean Health Sciences Literature (LILACS), ProQuest Dissertation and Thesis, and Google Scholar databases, with no temporal or language restrictions; duplicate articles were excluded using Endnote software (Clarivate Analytics^®^).

The descriptors used and the search strategies were: “Police”, “Burnout, Professional”, “Burnout, Psychological”, and “Occupational Stress” combined in the databases with Police, polices, Police Force, Law Enforcement Officers, Law Enforcement Officer, Police Officers, Police Officer, policing, Police Personnel, Professional Burnout, Occupational Burnout, Career Burnout, Occupational Stress, Psychological Burnout, Burn-out Syndrome, Burnout, burnouts, Burnout Syndrome, Burn-out, Psychological Burn-out, Occupational Stress, Occupational Stresses, Job Stress, Job Stresses, Work Stress, Work-related Stress, Work related Stress, Work-related Stresses, Workplace Stress, Workplace Stresses, Work Place Stress, Work Place Stresses, Professional Stress, Professional Stresses, Job-related Stress, Job-related Stresses, Workplace Bullying, Workplace Abuse, Workplace Abuses.

These descriptors were found in the Medical Subject Headings (MeSH), Health Sciences Descriptors (DeCS), and Emtree controlled vocabularies. In addition, synonyms and alternative terms were searched. The set of terms related to the police force was combined using the boolean operator AND and OR. The strategy described above was used in the databases according to their particularities. For the LILACS database, equivalent terms in Portuguese and Spanish were added. The complete strategies are available on the Open Science Framework (OSF)^(13)^, at the following link: https://osf.io/g3w9t/.

The search for records in the data sources was carried out on the Periodicals Portal of the Coordination for the Improvement of Higher Education Personnel (CAPES) through remote access to the content of the Federated Academic Community (CAFe), a resource funded by the University of São Paulo (USP).

### Results Analysis

The selection of studies was carried out by two independent reviewers in two phases. The first phase involved mapping the titles and abstracts of potentially relevant studies, as well as examining the selected materials that apparently met the inclusion criteria based on their abstracts. In the second stage, all selected articles were independently read, and those that deviated from the review’s objective and/or did not allow for the research question to be answered were excluded. Any disagreements were resolved by a third reviewer. Subsequently, the Rayyan software was used for data organization and extraction.

In presenting the results below, there is a flowchart that demonstrates the entirety of the searches and the selection process leading to the final corpus^(18)^. For the synthesis and better demonstration of the results, a descriptive structure was used to examine the text of each study. In the final stage, the results were compiled and communicated. The present review was registered in the OSF^(13)^ at https://osf.io/g3w9t/, doi: 10.17605/OSF.IO/G3W9T.

## RESULTS

**Figure 1:**
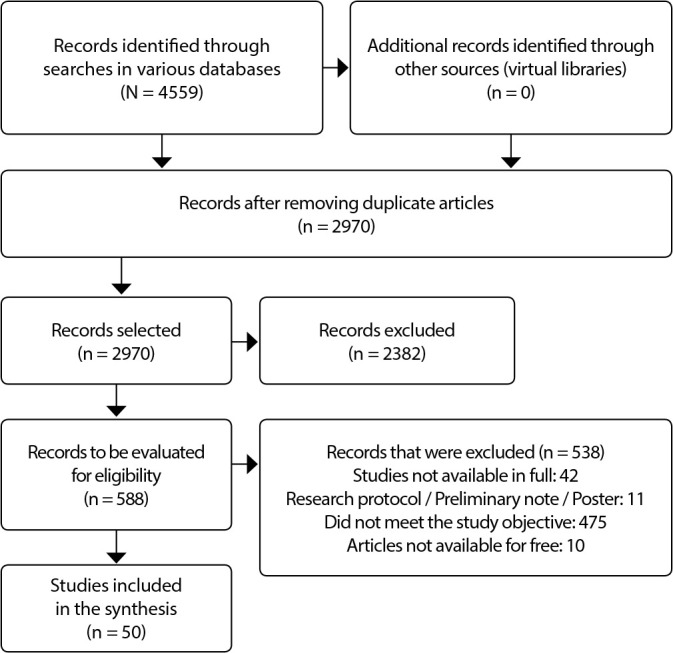
PRISMA-ScR Flowchart of Study Selection, Ribeirão Preto, São Paulo, Brazil, 2022

Initially, 4559 scientific publications were identified in the searched databases, with 419 in Medline/PubMed, 552 in Embase, 1012 in Scopus, 984 in Web of Science, 46 in Lilacs, 1028 in PsycINFO, 418 in ProQuest Dissertation and Thesis, and 100 in Google Scholar. 1589 were excluded, and after removing duplicates, 2970 were selected by title and abstract. Of these, 2382 did not meet the inclusion criteria, resulting in 588 articles for full reading and analysis. After reading the available articles, 50 remained in the final corpus of the study, as shown in the PRISMA Extension for Scoping Reviews (PRISMA-ScR) flowchart presented in Figure 1.

Regarding the languages of publication found, 44 studies were published in English, five in Portuguese, and one in Croatian. Concerning the publication year, the identified distribution was: 1983 (n=1)^(19)^, 1999 (n=1)^(20)^, 2002 (n=1)^(21)^, 2004 (n=1)^(22)^, 2006 (n=1)^(23)^, 2007 (n=2)^(24-25)^, 2010 (n=1)^(26)^, 2011 (n=3)^(27-29)^, 2012 (n=1)^(30)^, 2013 (n=1)^(31)^, 2014 (n=1)^(32)^, 2015 (n=3)^(33-35)^, 2016 (n=4)^(36-39)^, 2017 (n=4)^(40-43)^, 2018 (n=6)^(44-49)^, 2019 (n=5)^(50-54)^, 2020 (n=6)^(55-60)^, 2021 (n=2)^(61-62)^, and 2022 (n=6)^(63-68)^.

Regarding the 25 countries where the studies were conducted, the distribution was as follows: Brazil (n=6)^(37,40,44-46,62)^, United States (n=5)^(28-29,49,52,57)^, India (n=4)^(43,50,53,61)^, Israel (n=3)^(23-25)^, China (n=3)^(41,60,64)^, Portugal (n=3)^(31,58,68)^, Mexico (n=2)^(55,67)^, Spain (n=2)^(21,42)^, Germany (n=2)^(38,59)^, Sweden (n=2)^(30,39)^, Turkey (n=2)^(28,34)^, Poland (n=3)^(22,32-33)^, Norway (n=1)^(66)^, Netherlands (n=1)^(20)^, Finland (n=1)^(29)^, Nigeria (n=1)^(35)^, Jamaica (n=1)^(54)^, Croatia (n=1)^(56)^, Bulgaria (n=1)^(36)^, South Korea (n=1)^(49)^, Sri Lanka (n=1)^(48)^, Switzerland (n=1)^(51)^, Italy (n=1)^(63)^, Peru (n=1)^(65)^, and Canada (n=1)^(19)^. It is worth noting that a dissertation conducted in Portugal and two doctoral theses, one conducted in Canada and another in Turkey, were included due to the absence of publications from these studies in journals in the searched sources and because it is a scoping review in which such inclusion is permitted.

Overall, the studies indicated a scarcity of knowledge production on the subject in question, considering that, without limiting the time, the quantity included was small, considering the 50 studies from the 25 countries found over 40 years. It is noteworthy that the search was broad in various databases, so there was also no observed decrease in BS over the years and little planning of preventive strategies focused on the mental health of this population. Two identical articles published in different journals were identified, authored by the same authors, indicating a lack of ethical principles in research.

From the reading of the articles included in this review, factors were identified aiming to answer the objective of this review and to make contributions to the areas of Occupational Health, Public Health, and Mental Health; they are: age^(29,31)^, gender^(29,31,48,53,55,64)^, number of children^(44)^, education level^(29,35)^, completion of high school^(40)^, marital status^(19,35,65)^, post-traumatic stress^(24-25)^, hopelessness^(46,62)^, feelings of fatigue^(32)^, low-excitement negative emotions^(31,61)^, aggression^(31,61)^, anger^(31)^, emotional exhaustion^(31,47)^, insomnia^(66)^, insecurity^(61)^, variety and intensity of emotions^(40)^, depression^(62)^, suicidal ideation^(54,63)^, general stress^(19,25,30,39,58)^, psychological distress^(21,26)^, cynicism^(26,47)^, general distrust^(26)^, regular practice of physical exercise^(44)^, personality type^(22-42)^, low sociability^(5)^, low resilience^(58)^, overcommitment^(47)^, need for approval^(47)^, daytime sleepiness^(52)^, work-family conflict^(61)^, lack of social^(26,33,39)^ and organizational^(33,56,64)^ support, need to express positive emotions as part of police work^(40)^, physical aggression^(61)^, use of force^(20)^, organizational tensions^(34)^, low organizational commitment^(29,61)^, working in smaller agencies^(27)^, and organizational stressors^(27)^.

Other factors also identified included working in field service^(40)^, disciplinary punishment^(29)^, workload^(64)^, direct contact with supervisors^(64)^, shift schedules (irregular, rotating, fixed)^(29,52)^, shift characteristics (night, duration, frequency, working hours)^(52)^, increased night shifts^(52)^, exposure to media^(26,68)^, dependency on coworkers^(26,68)^, turnover intentions^(26,68)^, decreased work resources^(41)^, lack of organizational support^(33,56,64)^, years of service^(65)^, transfers to work locations without consent or prior notice^(5)^, conflict management^(20)^, civilian confrontations^(20)^, work experience^(31)^, reduced work resources^(47,56)^, organizational stressors^(24,47,58)^, low salaries^(24)^, lack of resources and work overload^(24)^, risk of victimization^(38)^, high work effort^(59)^, job stability^(35)^, increased work demands^(41)^, and police work burnout^(60)^.

Some occupational factors, such as law enforcement^(34)^, frequency of interaction with suspects and criminals^(40)^, being a sergeant^(40)^, dissatisfaction with the department^(5)^, and civilian confrontations^(20)^, which can lead to BS, have characteristics more related to police officers when compared to other professions.

It is worth noting that when BS is already established, factors such as loss of empathy^(67)^, avoidance behavior^(32)^, self-protective behavior^(38)^, decreased cortisol secretion^(57)^, favorable attitude towards violence^(38)^, poor sleep quality^(21-46)^, high morbidity and mortality rates^(46)^, functional aging^(46)^, poor self-perception of health status^(67)^, poor perception of diet quality^(67)^, irregular meals^(67)^, high body mass index^(67)^, among others, are evident.

## DISCUSSION

From this scoping review, it was possible to identify factors that contribute to burnout syndrome (BS) in police officers, given that this profession entails specific risks that favor the onset of this syndrome. It was also noted a lack of studies addressing this topic. Most of the studies were published in 2020 and 2022, with Brazil and the United States being the countries with the highest number of publications on this subject. The presence of BS in police officers and the factors that may contribute to its occurrence were identified as follows.

Historically, the Police had the function of protecting the assets and interests of a minority, at the expense of the less fortunate, with actions marked by repression, stigma, and prejudice. Some believed that the police were “inhuman” and “heartless”^(9)^.

It is known that the frequency of BS in this professional category is high, but authors have highlighted that individual personality factors may explain its development^(22,42)^. The activity of police officers falls mainly under physical and mental risks and has shown a high proven rate of non-communicable chronic diseases. In fact, among the professions governing Public Security, that of the police has shown higher rates of BS, poor sleep quality^(21,46)^, high morbidity and mortality rates, and functional aging^(46)^. The signs and symptoms of BS may be associated with decreased cortisol secretion^(57)^.

It is worth noting that, in this review, the male audience prevailed in all included studies. Gender mainly influenced how they dealt with stressful situations and, therefore, experienced BS differently^(53)^. Thus, men were at higher risk than women for the development of BS^(55)^. Among male police officers, BS was more prevalent among those with children^(44)^, and younger individuals had a positive association with such syndrome^(48)^. Significant differences were verified according to age and work experience^(31)^.

It is of utmost importance to mention that symptoms of post-traumatic stress disorder^(24-25)^, hopelessness^(46,62)^, depression^(62)^, suicidal ideation^(54,63)^, general stress^(25,30,39)^, psychological distress^(21,26)^, cynicism, intentions of job turnover, increased family protection, general distrust, and dependence on colleagues^(26)^ should be taken into consideration for the development of BS^(30,39)^, as they also contribute to the loss of empathy among individuals^(67)^. Higher levels of Burnout lead to avoidance behavior^(20)^.

Feelings of fatigue, low arousal negative emotions^(32)^, aggressiveness, anger, emotional exhaustion^(31)^, insomnia^(66)^, and feelings of insecurity can also influence behaviors of physical aggression^(61)^. A study conducted in the Netherlands pointed out significant correlations between burnout and the use of force^(20)^.

Other factors that have been predictors for BS include the variety and intensity of emotions and having completed high school education^(40)^. Additionally, the worst levels of the syndrome were found in individuals with poor self-perception of health status, poor perception of diet quality, irregular meals, and high body mass index^(67)^.

It is suggested that extreme work involvement and overcommitment may be related to the need for approval and the impossibility for employees to distance themselves from work, even during off-duty hours. Overcommitment was positively and significantly associated with cynicism and exhaustion, while professional efficacy showed an inverse association with overcommitment^(47)^.

A study with 573 German police officers showed that high work effort was associated with higher levels of burnout, while rewards at work and health-oriented leadership were associated with lower levels of such syndrome^(59)^. Research has identified variables such as age, education, work shift, role^(29)^, marital status, education, and job stability^(35)^ to be associated with the development of BS. Another study stated that sex and age were factors to be ruled out in the development of BS in evaluated police officers^(42)^.

Factors related to police work have been pointed out as favoring the emergence of BS; police officers have relatively low levels of emotional exhaustion and relatively high levels of depersonalization^(20)^. The results also showed that a high number of them had a high prevalence of burnout and a high level of mental exhaustion^(55)^. Other factors found in this population favoring BS include smaller police units, where these professionals experience significantly higher levels of exhaustion and stress compared to colleagues working in larger units^(27)^. It was also evident that organizational stressors were more pronounced when compared to operational stressors^(56)^. Predictors for BS were also identified, such as frequency of interaction with suspects and criminals, need to express positive emotions as part of police work, holding the rank of “sergeant,” and working in external services^(40)^.

A survey conducted with 538 employees of the Turkish national police showed that organizational and operational tensions in law enforcement were more strongly associated with levels of burnout^(34)^. BS was linked with organizational commitment and disciplinary actions^(29)^, gender^(29,31,64)^, workload, direct contact with supervisors^(64),^ work shift schedules (irregular, rotating, fixed), shift characteristics (night, duration, frequency, work hours), daytime sleepiness, increased night shifts^(52)^, media exposure, dependence on colleagues, intentions of turnover^(26-68)^, lack of organizational support^(33,56,64)^, lack of social support^(26,33,39)^, years of service, and marital status^(65)^.

Data collected in 2021 from 1,682 Portuguese police officers through individual surveys showed that BS was associated with a positive impact on work performance because when a person experiences burnout, the intention to leave the organization decreases^(68)^. Dissatisfaction with the corporation, low sociability, transfers to work locations without consent or prior notice can negatively impact the lives of police officers^(5)^.

A cross-sectional study using online questionnaires with 1,131 police officers showed that they experienced high levels of operational and organizational stress and had low resilience^(58)^. In Istanbul, police officers with BS showed a negative relationship with organizational commitment^(28)^; 358 Dutch police officers with BS, during conflict management, behaved differently in confrontations with civilians^(20)^.

In addition to coping with inhumane schedules, police officers work overtime on their days off to supplement their salary, making it challenging to establish a routine that promotes activities that effectively and harmoniously engage the body and mind^(5)^. Another significant factor is the limited work resources^(56)^. A study conducted with 223 police officers in 2007 observed that, for Norwegian police officers, the pressure between work and family was a predictor for all three dimensions of BS^(25)^.

A representative sample of 1010 Israeli police officers highlighted that during the violent Palestinian uprising, organizational stressors such as low salary, lack of resources, and overload were identified, with more than half of the police officers experiencing high levels of stress and burnout. However, despite the observed work stress, the officers reported high job satisfaction^(24)^. A survey conducted in 2020 in Croatia showed that surveyed police officers reported the highest support from colleagues, followed by family, while the lowest support was received from their superiors^(56)^. A cross-sectional study conducted with 1,742 German patrol police officers pointed out that police officers with BS reduced self-protective behavior, which in turn increased the risk of victimization. Depersonalization was positively associated with a favorable attitude towards violence, which was linked to violent victimization^(38)^.

A cross-sectional study involving a non-random sample of 1,223 officers indicated that the quantitative demands of BS had a negative effect on workplace commitment, organizational citizenship behaviors, and the health of the respondents^(61)^. However, another study pointed out that quantitative job demands were not significantly associated with burnout^(66)^. Findings from the capital of India revealed that the organizational interface and work-life balance significantly contributed to the development of BS^(43)^. Overall, emotional labor, role stressors, and emotional dissonance were related to higher levels of police burnout^(49)^. It’s also worth noting that the perceived lack of organizational support not only affected police job strain but also impacted job satisfactio^(60)^.

Using systematic random sampling, data collected from a survey conducted among 1,000 out of 3,000 police officers in the districts of Rohtak and Sonipat, in the state of Haryana, India, mentioned that conflict based on time, behavior, and family had positive associations with depersonalization exhaustion and reduced personal accomplishment exhaustion^(50)^. In Brasília, the capital of Brazil, a study involving 164 police officers demonstrated that job tasks and social characteristics negatively predicted burnout and, therefore, acted as protective variables^(62)^. It’s important to highlight that 234 Polish police officers prioritized extrinsic work values, and professional burnout was negatively correlated with intrinsic cognitive work values, which could interfere with work engagement^(51)^.

In a study conducted in Canada, 122 police officers appeared significantly more detached from non-occupational individuals in their lives. They also claimed that work-related exhaustion and stress could negatively impact marital relationships^(19)^.

A study conducted in Bulgaria in 2016 indicated that demographic characteristics did not influence the occurrence of BS, but there was a correlation between the level of burnout and the number of absences, need for medical assistance, and medication expenses. Officers affected by BS took more sick leaves, negatively affecting their remuneration, as they lost 3.1% of their annual salaries. Their medical expenses were three times higher, and their monthly medication expenses were 3.14 times higher than those of individuals without BS^(36)^.

A descriptive-quantitative study conducted with 25 civilian police officers working in the Intelligence Management of the State Secretariat of Security and Defense of Paraíba, Brazil, found signs of burnout, as medium levels of emotional exhaustion, low levels of depersonalization, and high levels of professional inefficacy were detected^(45)^. Another two-wave study involving 172 nurses and 273 police officers showed several common patterns in both samples, with increased work demands and decreased work resources in a highly demanding environment significantly increasing BS^(41)^.

A study conducted in Ceará, Brazil, evaluated the regular practice of physical exercises and found that both women and men engaged in physical activities at least twice a week and were at least in the initial phase of burnout^(44)^. It was mentioned that being married, having satisfactory infrastructure, frequent superior guidance, satisfactory support from senior officers, satisfactory subsidies, public service opportunities, team adequacy, satisfactory social status, and overall job satisfaction were negatively associated with BS^(48)^.

Unlike what has been mentioned so far, a study conducted in Brazil in 2016 found no incidence of BS among the surveyed police officers; however, it pointed out a prevalence of risk for its development^(37)^. The results underscored the importance of investing in the occupational health of police officers^(58)^. There is a need to review the working hours of this professional class to limit their physical and emotional exhaustion^(52)^. It’s important to emphasize that in some countries, there is an unknown landscape linked to this issue. Moreover, underreporting of BS illness may occur among workers in the Public Safety sector, a factor that could be better captured/estimated in future surveys. The urgency of guaranteeing the rights of Public Safety workers while reinforcing the agenda of mental health in this class is highlighted, a movement that should be captured by future production.

A small number of studies associated with the topic in question were identified in the literature. It was noticed that, after a period of 40 years of study, the same questions from the initial studies remain, indicating that there has been no advancement in terms of solving BS within this public. Conversely, the increase in the number of studies on this topic emphasizes the recognition of its importance.

### Study limitations

This study presents some limitations that should be taken into consideration. Firstly, it is important to highlight that relevant research may not have been published in the academic journals searched, and government documents and proceedings of scientific events that could contain pertinent information may not have been included in the analysis. Additionally, scoping reviews do not include a critical evaluation of the methodologies employed in the included studies, as different instruments were used in data measurement. We acknowledge that this methodological diversity may have impacted the quality of the studies presented in this review. However, we emphasize that more research is needed for a deeper investigation into this syndrome and its relationship with the work of police officers.

### Contributions to the Field of Public Safety, Occupational Health, Mental Health, and Public Health

This article aims to contribute to the understanding of factors contributing to burnout, having a significant impact on the areas of Public Safety, Occupational Health, Mental Health, and Public Health. Its contribution to knowledge production on this topic is remarkable, advancing understanding and providing insights for future research and policies aimed at this population. In addition to providing a solid foundation for subsequent studies, this article suggests the need for measures and policies specifically targeting police officers, with the aim of promoting the physical and mental health of these professionals. Interventional actions are essential to ensure the well-being of police officers and prevent the onset of burnout. It is crucial to develop projects aimed at improving the quality of work life for police officers, as well as encouraging scientific research to assess the results and impacts of these initiatives in different regional contexts. Therefore, strategies and public policies aimed at preventing burnout among police officers are urgently needed and should be implemented.

## CONCLUSIONS

There was a predominance of research conducted in Brazil and the United States, with the majority involving male professionals. Labor characteristics favoring the onset of BS among the surveyed population were identified, as well as factors influencing its occurrence. Despite observing that some countries have better working conditions for police officers in terms of safety, it was found that they are still affected by this syndrome.

Several factors contributing to the onset of BS were identified, including individual and work-related characteristics, some of which are specific to police officers when compared to other professions. The results of the studies selected in this review revealed the complexity of this phenomenon, emphasizing the importance of continuing to discuss the labor aspects of this population in the fields of Mental Health and Occupational Health, both by public managers and professional associations.
