# Supplementary material for: Factors associated with Burnout Syndrome in police officers: a scoping review
Source: Rev Bras Enferm. 2024 Jul 15;77(2):e20230444. doi: 10.1590/0034-7167-2023-0444 (PMC11259444; doi:10.1590/0034-7167-2023-0444)
Supplement: Translation

# Translation: Fatores associados à Síndrome de Burnout em policiais: revisão de escopo

**DOI:** 10.1590/0034-7167-2023-0444pt

**Authors:** Beatriz Maria dos Santos Santiago Ribeiro, Fabio Scorsolini-Comin, Maria Lucia do Carmo Cruz Robazzi, Sérgio Valverde Marques dos Santos, Fábio de Souza Terra, Rita de Cassia de Marchi Barcellos Dalri

**Affiliations:** I Universidade de São Paulo. Ribeirão Preto, São Paulo, Brasil; II Universidade do Estado de Minas Gerais. Passos, Minas Gerais, Brasil; III Universidade Federal de Alfenas. Alfenas, Minas Gerais, Brasil

**Keywords:** Segurança Pública, Esgotamento Psicológico, Policiais, Saúde Ocupacional, Enfermagem

## Abstract

**Objetivos:**

sintetizar os estudos que abordam os fatores associados à síndrome de *burnout* (SB) em policiais.

**Métodos:**

revisão de escopo, sem restrição temporal e de idiomas, com exportação para o *EndNote*, suprimindo os duplicados, e exportados para o aplicativo *Rayyan*, para organização, seleção dos artigos e extração dos dados.

**Resultados:**

foram identificadas 4559 publicações, com inclusão de 50 estudos. Houve predominância de pesquisas realizadas no Brasil e nos Estados Unidos. Alguns fatores laborais foram mais relacionados aos policiais quando comparados com outras profissões, como a aplicação da lei, frequência de interação com suspeitos e criminosos, ser cabo, insatisfação com a corporação e confrontos com civis.

**Conclusões:**

certos aspectos laborais contribuem para a SB, mesmo em países com melhores condições de trabalho na Segurança Pública. Recomenda-se priorizar ações de promoção da saúde para esses profissionais.

## INTRODUÇÃO

A síndrome de *burnout* (SB) é causada pelo esgotamento físico e mental, que pode resultar em comportamentos negativos^(1)^. A primeira dimensão da SB é a exaustão emocional, marcada pela ausência de energia, entusiasmo e sentimento de esgotamento de soluções. É possível surgir um sentimento de frustração, sendo que os trabalhadores podem se sentir esgotados para o atendimento ao cliente, percebendo que sua atuação não é tão competente como antes^(1)^.

A segunda dimensão é a despersonalização, situação em que o profissional trata as pessoas e a organização como objetos, podendo desenvolver insensibilidade emocional. A terceira dimensão é a baixa realização profissional, caracterizada como uma tendência do trabalhador autoavaliar-se de forma negativa, sentindo-se infeliz consigo mesmo e insatisfeito com sua atuação profissional, ocorrendo um declínio do sentimento de competência e êxito e, também, uma diminuição da capacidade de interagir com os outros^(1)^. Investigar a SB permite a execução do conceito de integralidade das ações de Saúde do Trabalhador.

No cotidiano de trabalho, é muito comum a exposição simultânea aos fatores de risco para o desenvolvimento da SB. Como ela afeta a saúde do trabalhador, pode ser concedido afastamento, porém o adoecimento associado deve ser incluído como diagnóstico secundário. O reconhecimento da SB no ambiente laboral deve ser feito a partir da Comunicação de Acidente de Trabalho (CAT). Tal síndrome passou a ser enquadrada, no ano de 2022, na Classificação Internacional de Doenças (CID 11) como QD85, no capítulo intitulado Problemas Associados ao Emprego e Desemprego; estando fora do capítulo referente aos transtornos mentais, comportamentais ou do neurodesenvolvimento presente na CID 10^(2)^.

O trabalho dos policiais é frequentemente arriscado e insalubre. Na sua atuação profissional, há fatores inerentes ao desenvolvimento da SB. Na maioria das vezes, eles não podem manifestar sentimentos como prazer, inquietude e medo, com o risco de serem vistos como fracos e covardes. Os recém-formados são submetidos a uma avaliação pelo grupo de superiores, sendo que essa situação pode ser fonte de ansiedade, pressão e sofrimento^(3)^, podendo trazer agravos à sua saúde mental.

A polícia assegura o cumprimento da lei, por meio da preservação da ordem pública, preza pela satisfação das necessidades de segurança da comunidade e auxilia-a de modo preventivo^(4)^. Como um mecanismo de defesa, alguns policiais criam uma postura rígida não só na instituição na qual atuam, como nas relações sociais^(3)^. No entanto, esse trabalho exige que esse profissional tenha resiliência e boa capacidade de adaptação para lidar e superar os possíveis efeitos decorrentes do seu cotidiano, mas nem sempre isso ocorre com todos os policiais^(5)^.

Os policiais estão expostos às situações de perigo em sua rotina, quando sua própria vida é colocada em risco, e essa tensão também se estende aos momentos de folga. Estudos realizados com policiais brasileiros demonstraram estresse e falta de reconhecimento pelo esforço realizado^(6-7)^, sentimento de frustração, inutilidade e improdutividade, condições de trabalho inadequadas, representadas pela falta de equipamentos, escassez de instalações físicas e de recursos humanos insuficientes^(8)^, influenciando o desgaste da saúde física e psíquica desses profissionais^(9)^.

Frente ao exposto até então, observou-se a necessidade de pesquisar a SB no trabalho na área da Segurança Pública, em especial nos policiais, pois é uma profissão marcada por riscos iminentes e estresse, enfatizando os fatores relacionados às causas e sintomas que podem levá-los à SB. Este estudo pode contribuir para a saúde e segurança desses trabalhadores, refletindo em melhores condições de segurança para a população, bem como na melhoria da qualidade de vida no trabalho desses profissionais. Espera-se também que a presente revisão possa contribuir para o desenvolvimento de medidas preventivas e ações de promoção de saúde voltadas a esse público.

A pergunta de pesquisa que norteou este estudo foi: quais as evidências científicas que abordam policiais acometidos pela SB?

## OBJETIVOS

Sintetizar os estudos que abordam os fatores associados à síndrome de *burnout* (SB) em policiais

## MÉTODOS

### Desenho e período do estudo

Trata-se de uma revisão de escopo, cujo objetivo é mapear as principais definições e evidências científicas disponíveis sobre uma determinada área/tema^(10)^. As buscas foram realizadas em dezembro de 2022.

### Critérios de inclusão

Os critérios de inclusão adotados foram: estudos escritos em qualquer idioma, sem restrição temporal e que respondessem à pergunta de pesquisa. Incluíram-se estudos com diferentes níveis de evidência, permitindo conhecer de modo mais aproximado o estado da arte a respeito de uma dada questão^(11)^. Com o propósito de garantir a integridade deste estudo e seu rigor metodológico, seguiu-se os cinco critérios preconizados no protocolo *Joanna Briggs Institute* (JBI): estabelecimento da questão de pesquisa, identificação de estudos, seleção e inclusão de estudos e organização dos estudos selecionados e, por fim, a interpretação, a análise e a compilação dos resultados^(10)^.

### Protocolo do estudo

Foi verificada a existência de revisões de escopo de pesquisa semelhantes nas plataformas: *International Prospective Register of Systematic Reviews* (PROSPERO)^(12)^, *Open Science Framework* (OSF)^(13)^, *The Cochrane Library* ^(14)^, *JBI Clinical Online Network of Evidence for Care and Therapeutics* (COnNECT+)^(15)^, e *Database of Abstracts of Reviews of Effects* (DARE)^(16)^. Os resultados evidenciaram a inexistência de revisões com objetivo e conteúdos similares ao deste estudo.

Utilizou-se a estratégia PCC (acrônimo de P: População = Policiais, C: Conceito = Síndrome de *Burnout*, C: Qualquer contexto = Policiais acometidos pela Síndrome de *Burnout*), para a elaboração da questão norteadora do estudo, ou seja, quais as evidências científicas que abordam sobre policiais acometidos pela SB?

Para gerenciamento dos registros foi utilizado o *software Rayyan*, indicado para revisões sistemáticas e meta-análises^(17)^.

As buscas foram realizadas nas bases/bibliotecas *Medline/PubMed, EMBASE, Scopus, Web of Science, PsycINFO*, Literatura Latino-Americana e do Caribe em Ciências da Saúde (LILACS), *ProQuest Dissertation and Thesis* e *Google Scholar*, sem restrição temporal e de idiomas; foram excluídos os artigos duplicados mediante o uso de *software Endnote* (*Clarivate Analytics^®^*).

Os descritores utilizados e as estratégias de busca foram: “Police”, “Burnout, Professional”, “Burnout, Psychological” e “Occupational Stress” combinados nas bases com *Police, polices, Police Force, Law Enforcement Officers, Law Enforcement Officer, Police Officers, Police Officer, policing, Police Personnel, Professional Burnout, Occupational Burnout, Career Burnout, Occupational Stress, Psychological Burnout, Burn-out Syndrome, Burnout, burnouts, Burnout Syndrome, Burn-out, Psychological Burn-out, Occupational Stress, Occupational Stresses, Job Stress, Job Stresses, Work Stress, Work-related Stress, Work related Stress, Work-related Stresses, Workplace Stress, Workplace Stresses, Work Place Stress, Work Place Stresses, Professional Stress, Professional Stresses, Job-related Stress, Job-related Stresses, Workplace Bullying, Workplace Abuse, Workplace Abuses*.

Esses descritores foram encontrados nos vocabulários controlados *Medical Subject Headings* (MeSH), Descritores em Ciências da Saúde (DeCS) e *Emtree*. Além disso, foram pesquisados sinônimos e termos alternativos. Combinou-se o conjunto de termos relacionados à força policial utilizando-se o operador booleano AND e OR. A estratégia descrita anteriormente foi utilizada nas bases de dados de acordo com as particularidades de cada uma delas. Para a base de dados LILACS foram acrescentados os termos equivalentes em português e espanhol. As estratégias completas estão disponíveis no *Open Science Framework* (OSF)^(13)^, no link: https://osf.io/g3w9t/.

A busca dos registros nas fontes de dados foi efetuada no Portal de Periódicos da Coordenação de Aperfeiçoamento de Pessoal de Nível Superior (CAPES) por meio de acesso remoto ao conteúdo da Comunidade Acadêmica Federada (CAFe), recurso financiado pela Universidade de São Paulo (USP).

### Análise dos resultados

A seleção dos estudos foi realizada por dois juízes independentes, em duas fases. A primeira correspondeu ao mapeamento dos títulos e dos resumos de estudos potencialmente relevantes, bem como à observação dos materiais selecionados que aparentemente cumpriam os critérios de inclusão, baseados em seus resumos. Já na segunda etapa, ocorreu a leitura de todos os artigos selecionados, de forma independente, sendo excluídos os que se distanciavam do objetivo da revisão e/ou que não permitiam responder à questão de pesquisa. As possíveis discordâncias foram solucionadas por um terceiro juiz. Posteriormente, para organização e extração dos dados, foi utilizado o *software Rayyan*.

Na apresentação dos resultados na sequência, há um fluxograma que demonstra a totalidade das buscas e o percurso de seleção e composição do corpus final^(18)^. Para a síntese e melhor demonstração dos resultados, utilizou-se uma estrutura descritiva para examinar o texto de cada estudo. Na etapa final, realizaram-se a compilação e a comunicação dos resultados. A presente revisão foi registrada na OSF^(13)^ em https://osf.io/g3w9t/, doi 10.17605/OSF.IO/G3W9T.

## RESULTADOS

Foram identificadas inicialmente 4559 publicações científicas nas bases de dados pesquisadas, sendo 419 na *Medline/PubMed*, 552 na *Embase*, 1012 na Scopus, 984 na *Web of Science*, 46 na Lilacs, 1028 na *PsycINFO*, 418 na *ProQuest Dissertation and Thesis* e 100 no *Google Scholar*. Foram excluídas 1589 e após a remoção das duplicadas, restaram 2970 selecionadas por título e resumo. Destas, 2382 não atendiam aos critérios de inclusão, resultando em 588 artigos para a leitura e análise completa. A partir da leitura dos artigos disponíveis, permaneceram 50 no corpus final do estudo, conforme fluxograma *PRISMA Extension for Scoping Reviews* (PRISMA-ScR) apresentado na Figura 1.

**Figura 1:** Fluxograma PRISMA-ScR de seleção dos estudos, Ribeirão Preto, São Paulo, Brasil, 2022

Em relação aos idiomas de publicação encontrados, 44 estudos foram publicados em inglês, cinco em português e um em croata. No que diz respeito ao ano de publicação, a distribuição identificada foi: 1983 (n=1)^(19)^, 1999 (n=1)^(20)^, 2002 (n=1)^(21)^, 2004 (n=1)^(22)^, 2006 (n=1)^(23)^, 2007 (n=2)^(24-25)^, 2010 (n=1)^(26)^, 2011 (n=3)^(27-29)^, 2012 (n=1)^(30)^, 2013 (n=1)^(31)^, 2014 (n=1)^(32)^, 2015 (n=3)^(33-35)^, 2016 (n=4)^(36-39)^, 2017 (n=4)^(40-43)^, 2018 (n=6)^(44-49)^, 2019 (n=5)^(50-54)^, 2020 (n=6)^(55-60)^, 2021 (n=2)^(61-62)^ e 2022 (n=6)^(63-68)^.

Quanto aos 25 países de desenvolvimento dos estudos, a distribuição foi a seguinte: Brasil (n=6)^(37,40,44-46,62)^, Estados Unidos (n=5)^(28-29,49,52,57)^, Índia (n=4)^(43,50,53,61)^, Israel (n=3)^(23-25)^, China (n=3)^(41,60,64)^, Portugal (n=3)^(31,58,68)^, México (n=2)^(55,67)^, Espanha (n=2)^(21,42)^, Alemanha (n=2)^(38,59)^, Suécia (n=2)^(30,39)^, Turquia (n=2)^(28,34)^, Polônia (n=3)^(22,32-33)^, Noruega (n=1)^(66)^, Holanda (n=1)^(20)^, Finlândia (n=1)^(29)^, Nigéria (n=1)^(35)^, Jamaica (n=1)^(54)^, Croácia (n=1)^(56)^, Bulgária (n=1)^(36)^, Coréia do Sul (n=1)^(49)^, Sri Lanka (n=1)^(48)^, Suíça (n=1)^(51)^, Itália (n=1)^(63)^, Peru (n=1)^(65)^ e Canadá (n=1)^(19)^. Ressalta-se que incluiu-se uma dissertação realizada em Portugal e duas teses de doutorado, uma realizada no Canadá e outra na Turquia, devido ao fato de não haver publicação proveniente destes estudos em periódicos nas fontes pesquisadas e por se tratar de uma revisão de escopo na qual é permitido.

De modo geral, os estudos apontaram que existe escassez de produção de conhecimento na temática em questão, haja vista que, por não ter limitado o tempo, a quantidade incluída foi pequena, considerando-se os 50 estudos oriundos dos 25 países encontrados ao longo de 40 anos. Ressalta-se que a busca foi ampla em diversas bases de dados, portanto tampouco se observou a diminuição da SB com o passar dos anos e o pouco planejamento de estratégias preventivas voltadas à saúde mental deste público. Identificaram-se dois artigos idênticos publicados em diferentes periódicos, sendo a autoria dos mesmos autores, denotando-se a falta de princípios éticos em pesquisa.

A partir da leitura dos artigos incluídos nesta revisão, identificou-se fatores visando responder ao objetivo desta revisão e promover contribuições para as áreas da Saúde do Trabalhador, Saúde Pública e Saúde Mental; sendo: idade^(29,31)^, gênero^(29,31,48,53,55,64)^, quantidade de filhos^(44)^, escolaridade^(29,35)^, possuir ensino médio completo^(40)^, estado civil^(19,35,65)^, estresse pós-traumático^(24-25)^, desesperança^(46,62)^, sentimentos de fadiga^(32)^, emoções negativas de baixa excitação, agressividade^(31,61)^, raiva^(31)^, exaustão emocional^(31,47)^, insônia^(66)^, insegurança^(61)^, variedade e intensidade das emoções^(40)^, depressão^(62)^, ideação suicida^(54,63)^, estresse geral^(19,25,30,39,58)^, mal-estar psicológico^(21,26)^, cinismo^(26,47)^, desconfiança geral^(26)^, prática regular de exercício físico^(44)^, tipo de personalidade^(22-42)^, baixo nível de sociabilidade^(5)^, baixa resiliência^(58)^,supercomprometimento^(47)^, necessidade de aprovação^(47)^, sonolência diurna^(52)^,pressão entre trabalho e família, trabalho-casa^(61)^, falta de apoio social^(26,33,39)^ e organizacional^(33,56,64)^, necessidade de expressar emoções positivas como parte do trabalho policial^(40)^, agressão física^(61)^, uso da força^(20)^, tensões organizacionais^(34)^, pouco comprometimento organizacional^(29,61)^, trabalhar em agências menores^(27)^ e estressores organizacionais^(27)^.

Outros fatores também identificados foram atuar no serviço externo^(40)^, punição disciplinar^(29)^, carga de trabalho^(64)^, contato direto com supervisores^(64)^, horários de turno (irregulares, rotativos, fixos)^(29,52)^, características do turno (noite, duração, frequência, horário de trabalho)^(52)^, aumento de turnos noturnos^(52)^, exposição à mídia^(26,68)^, dependência de colegas de trabalho^(26,68)^, intenções de rotatividade^(26,68)^, diminuição dos recursos de trabalho^(41)^, falta de apoio organizacional^(33,56,64)^, anos de serviço^(65)^, transferências de local de trabalho sem consentimento ou aviso prévio^(5)^, gestão de conflitos^(20)^, confrontos com civis^(20)^, experiência de trabalho^(31)^, recursos reduzidos de trabalho^(47,56)^, estressores organizacionais^(24,47,58)^, baixos salários^(24)^, falta de recursos e sobrecarga de trabalho^(24)^, risco de vitimização^(38)^, alto esforço de trabalho^(59)^, estabilidade no trabalho^(35)^, aumento de demandas de trabalho^(41)^ e desgaste do trabalho policial^(60)^.

Alguns fatores laborais, como aplicação da lei^(34)^, frequência de interação com suspeitos e criminosos^(40)^, ser cabo^(40)^, insatisfação com a corporação^(5)^ e confrontos com civis^(20)^ ,que podem levar à SB, têm características relacionadas mais com os policiais se comparados com outras profissões.

Ressalta-se que quando a SB já está instalada evidencia-se fatores como perda da empatia^(67)^, comportamento de evitação^(32)^, comportamento de autoproteção^(38)^, diminuição da secreção de cortisol^(57)^, atitude favorável em relação à violência^(38)^, baixa qualidade do sono^(21-46)^, elevadas taxas de morbimortalidade^(46)^, envelhecimento funcional^(46)^, autopercepção ruim do estado de saúde^(67)^, má percepção da qualidade da dieta^(67)^, refeições irregulares^(67)^, índice de massa corporal elevado^(67)^, entre outros.

## DISCUSSÃO

A partir desta revisão de escopo, foi possível identificar fatores que levam à SB em policiais, visto que essa profissão possui riscos específicos que favorecem o surgimento dessa síndrome. Permitiu-se identificar ainda a carência de estudos que abordam esse tema. A maior parte dos estudos foi publicada em 2020 e 2022, sendo o Brasil e os Estados Unidos os países com maior número de publicações nessa temática. Identificou-se a presença da SB em policiais e os fatores que podem contribuir para sua ocorrência, disponibilizados a seguir.

Historicamente, a Polícia tinha como função proteger os bens e interesses de uma minoria, em detrimento dos menos favorecidos, com ações marcadas por repressões, estigmas e preconceitos. Alguns acreditavam que a polícia era “desumana” e “sem coração”^(9)^.

É sabido que a frequência da SB nessa categoria profissional é alta, porém autores destacaram que fatores individuais de personalidade podem explicar seu desenvolvimento^(22,42)^. A atividade dos policiais enquadrou-se em riscos principalmente de ordem física e mental e apresentou alto índice comprovado de doenças crônicas não transmissíveis. Aliás, dentre as profissões que regem a Segurança Pública, a do policial apresentou maiores taxas de SB, baixa qualidade do sono^(21,46)^, elevadas taxas de morbimortalidade e de envelhecimento funcional^(46)^. Os sinais e sintomas da SB podem estar associados à diminuição da secreção de cortisol^(57)^.

Ressalta-se que, nesta revisão, em todos os estudos incluídos, prevaleceu o público masculino. O sexo influenciou principalmente na forma como lidaram com situações estressantes e, portanto, experimentaram a SB diferentemente^(53)^. Assim, os homens apresentaram maior risco do que as mulheres para o desenvolvimento da SB^(55)^. Entre os policiais do sexo masculino, a SB foi mais prevalente entre os que tinham filhos^(44)^ e os mais jovens tiveram associação positiva com tal síndrome^(48)^. Diferenças significativas foram verificadas de acordo com a idade e a experiência de trabalho^(31)^.

É de extrema importância mencionar que sintomas de estresse pós-traumático^(24-25)^, desesperança^(46,62)^, depressão^(62)^, ideação suicida^(54,63)^, estresse geral^(25,30,39)^, mal-estar psicológico^(21,26)^, cinismo, intenções de rotatividade laboral, aumento da proteção da família, desconfiança geral e dependência de colegas^(26)^ devem ser levados em consideração para o desenvolvimento da SB^(30,39)^, além de contribuírem para a perda da empatia entre as pessoas^(67)^. Os níveis mais elevados de SB levam ao comportamento de evitação^(20)^.

Sentimentos de fadiga, emoções negativas de baixa excitação^(32)^, agressividade, raiva, exaustão emocional^(31)^, insônia^(66)^ e também o sentimento de insegurança podem influenciar nos comportamentos de agressão física^(61)^. Um estudo realizado na Holanda apontou correlações significativas entre *burnout* e o uso da força^(20)^.

Outros fatores que se mostraram preditores para a SB foram a variedade e intensidade das emoções e possuir ensino médio completo^(40)^. Além disso, os piores níveis da síndrome estavam presentes em pessoas com autopercepção ruim do estado de saúde, má percepção da qualidade da dieta, sem refeições regulares e índice de massa corporal elevado^(67)^.

Sugere-se que o envolvimento extremo no trabalho e o supercomprometimento podem estar relacionados à necessidade de aprovação e à impossibilidade de os servidores afastarem-se do trabalho, mesmo em regime de folga. O supercomprometimento foi positivo e significativamente associado ao cinismo e à exaustão, enquanto a eficácia profissional mostrou uma associação inversa com supercomprometimento^(47)^.

Um estudo realizado com 573 policiais alemães mostrou que o alto esforço de trabalho associou-se aos níveis mais altos de *burnout,* enquanto as recompensas no trabalho e a liderança voltada para a saúde foram associadas aos níveis mais baixos de tal síndrome^(59)^. Pesquisas identificaram que variáveis como a idade, educação, turno de trabalho, função^(29)^, estado civil, escolaridade e estabilidade no trabalho^(35)^ associaram-se ao desenvolvimento da SB. Já outro estudo afirmou que o sexo e a idade eram fatores a serem descartados no desenvolvimento da SB em policiais avaliados^(42)^.

Fatores relacionados ao trabalho do policial foram apontados como favorecedores para o surgimento da SB; os policiais têm níveis relativamente baixos de exaustão emocional e níveis relativamente altos de despersonalização^(20)^. Os resultados mostraram também que um elevado número deles apresentou alta prevalência de *burnout* e um alto nível de exaustão mental^(55)^. Outros fatores constatados nesse público favorecedores para a SB foram as unidades policiais menores, nas quais esses profissionais experimentam níveis significativamente mais altos de esgotamento e estresse comparados com colegas que trabalham em unidades maiores^(27)^. Também evidenciou-se que os estressores organizacionais foram mais expressivos quando comparados com os operacionais^(56)^. Foram identificados ainda preditores para a SB como frequência de interação com suspeitos e criminosos, necessidade de expressar emoções positivas como parte do trabalho policial, exercer a função de “cabo” e atuar no serviço externo^(40)^.

Uma pesquisa realizada com 538 funcionários da polícia nacional turca mostrou que as tensões organizacionais e operacionais na aplicação da lei foram mais associadas aos níveis da SB^(34)^. A SB foi associada ao comprometimento organizacional e à punição disciplinar^(29)^, ao sexo^(29,31,64)^, à carga de trabalho, se tinham contato direto com supervisores^(64)^, horários dos turnos de trabalho (irregulares, rotativos, fixos), características do turno (noite, duração, frequência, horário de trabalho), sonolência diurna, aumento de turnos noturnos^(52)^, exposição à mídia, dependência de colegas de trabalho, intenções de rotatividade^(26-68)^, falta de apoio organizacional^(33,56,64)^, falta de apoio social^(26,33,39)^, anos de serviço e estado civil^(65)^.

Dados recolhidos em 2021 entre 1.682 policiais portugueses, por meio de inquéritos individuais, mostraram que a SB associou-se à interferência positiva no desempenho laboral, pois quando uma pessoa sofre de *burnout* diminui a intenção de sair da organização^(68)^. Insatisfação com a corporação, baixo nível de sociabilidade, transferências de local de trabalho sem consentimento ou aviso prévio podem interferir negativamente na vida dos policiais^(5)^.

Um estudo transversal que utilizou questionários online em 1.131 policiais evidenciou que eles apresentaram alto nível de estresse operacional e organizacional e baixo enfrentamento resiliente^(58)^. Em Istambul, policiais com SB apresentaram relação negativa com o comprometimento organizacional^(28)^; 358 policiais holandeses com SB, durante gestão de conflitos, agiram de forma diferente em confrontos com civis^(20)^.

Além de lidarem com escalas desumanas, os policiais submetem-se a horas extras em suas folgas para complementação do salário, o que torna difícil a realização de uma rotina que preconize atividades que trabalhem de modo efetivo e harmônico com o corpo e a mente^(5)^. Outro fator impactante são os recursos reduzidos de trabalho^(56)^. Um estudo realizado com 223 policiais em 2007 observou que, para policiais noruegueses, a pressão entre trabalho e família foi um preditor para todas as três dimensões da SB^(25)^.

Uma amostra representativa de 1010 policiais israelenses destacou que durante a revolta violenta palestina foram identificados estressores organizacionais como baixo salário, falta de recursos e sobrecarga, nos quais mais da metade dos policiais tiveram níveis altos de estresse e *burnout.* Mas mesmo com todo o estresse laboral verificado, a avaliação dos oficiais demonstrou alta satisfação em seu trabalho^(24)^. Uma pesquisa realizada em 2020 na Croácia apresentou que os policiais pesquisados relataram ter o maior apoio dos colegas, seguidos pela família, enquanto o menor apoio era recebido de seus superiores^(56)^. Um estudo transversal realizado com 1.742 policiais alemães de patrulha apontou que os policiais com SB reduziram comportamento de autoproteção, o que, por sua vez, aumentou o risco de vitimização. A despersonalização estava positivamente associada a uma atitude favorável em relação à violência, que estava associada à vitimização violenta^(38)^.

Uma pesquisa transversal que trabalhou com uma amostra não aleatória de 1.223 oficiais indicou que as demandas quantitativas de SB tiveram um efeito negativo no comprometimento com o local de trabalho, nos comportamentos de cidadania organizacional e na saúde dos pesquisados^(61)^. Já outro estudo apontou que as demandas quantitativas do trabalho não tiveram associação significativa com *burnout* ^(66)^. Na capital da Índia, achados revelaram que a interface organizacional e trabalho-casa contribuíram positiva e significativamente para o desenvolvimento da SB^(43)^. No geral, o trabalho emocional, os estressores de papéis e a dissonância emocional estavam relacionados ao maior esgotamento dos policiais^(49)^. Ainda destaca-se que a falta de suporte organizacional percebido não afetou apenas o desgaste do trabalho policial, mas também afetou a satisfação no trabalho^(60)^.

Utilizando-se de amostragem aleatória sistemática, os dados coletados em uma pesquisa realizada entre 1.000 dos 3.000 policiais nos distritos de Rohtak e Sonipat, no estado de Haryana, na Índia, mencionaram que o conflito baseado no tempo, no comportamento e na família teve associações positivas com o esgotamento da despersonalização e com a redução do senso de esgotamento da realização pessoal^(50)^. Em Brasília, capital do Brasil, um estudo realizado com 164 policiais demonstrou que a tarefa laboral e as características sociais predizem negativamente o *burnout* e, portanto, foram variáveis protetoras^(62)^. É importante destacar que 234 policiais poloneses deram maior prioridade aos valores extrínsecos do trabalho e que o esgotamento profissional foi negativamente correlacionado com os valores cognitivos intrínsecos do trabalho, os quais podem ter interferência no engajamento laboral^(51)^.

Em uma pesquisa realizada no Canadá, 122 policiais apareceram significativamente mais despersonalizados com pessoas em suas vidas não ocupacionais. Afirmaram ainda que o esgotamento e o estresse relacionados ao trabalho podem ter um efeito negativo sobre o relacionamento conjugal^(19)^.

Uma pesquisa realizada em 2016 na Bulgária apontou que as características demográficas não influenciaram na ocorrência da SB, mas houve correlação entre o nível de *burnout* e o número de afastamentos, necessidade de auxílio médico e gastos com medicamentos. Os oficiais acometidos por SB tiraram mais licenças médicas e isso afetou negativamente sua remuneração, pois perderam 3,1% de seus salários anuais. Suas despesas com serviços médicos foram três vezes maiores e seus gastos mensais com medicamentos foram 3,14 vezes maiores do que os das pessoas sem a SB^(36)^.

Um estudo descritivo-quantitativo realizado com 25 policiais civis que atuavam na Gerência de Inteligência da Secretaria de Estado da Segurança e da Defesa Social do estado da Paraíba, Brasil, constatou traços de *burnout*, uma vez que detectou-se a exaustão emocional em nível médio, despersonalização em nível baixo e profissional em nível alto^(45)^. Já outro estudo de duas ondas com 172 enfermeiros e 273 policiais mostrou vários padrões comuns em ambas as amostras, sendo que o aumento de demandas de trabalho e a diminuição dos recursos de trabalho em um ambiente altamente exigente mostrou um aumento significativo na SB^(41)^.

Um estudo realizado no Ceará, Brasil, avaliou a prática regular de exercícios físicos e observou-se que tanto mulheres quanto homens praticavam atividades físicas pelo menos duas vezes por semana e encontravam-se, pelo menos, na fase inicial do *burnout* ^(44)^. Citou-se que ser casado, ter infraestrutura satisfatória, orientação superior frequente, apoio de oficial superior satisfatório, subsídios satisfatórios, oportunidade de servir o público, adequação da equipe, status social satisfatório e satisfação geral no trabalho apresentaram associações negativas com a SB^(48)^.

Diferente do citado até o momento, um estudo realizado em 2016 no Brasil verificou que não há incidência da SB entre os policiais pesquisados, contudo, apontou que encontrou-se prevalência de risco para seu desenvolvimento^(37)^. Os resultados reforçaram a importância de se investir na saúde ocupacional dos policiais^(58)^. Há necessidade de se rever a carga horária dessa classe profissional para limitar seu esgotamento físico e emocional^(52)^. É importante enfatizar que em alguns países têm-se um panorama desconhecido vinculado a essa temática. Ainda, pode ser que ocorra a subnotificação do adoecimento pela SB nos trabalhadores que atuam na área da Segurança Pública, fator este que pode ser mais bem capturado/estimado em levantamentos futuros. Ressalta-se a premência de garantir os direitos dos trabalhadores da Segurança Pública ao mesmo tempo em que se reforça a pauta da saúde mental nessa classe, movimento que deve ser capturado pela produção vindoura.

Identificou-se uma quantidade pequena de estudos associados à temática em questão disponibilizados na literatura. Percebeu-se que, após um período de 40 anos de estudo, permanecem os mesmos questionamentos dos estudos iniciais, indicando que não se avançou em termos da solução da SB junto a esse público. Em contrapartida, a ampliação do número de estudos sobre essa temática enfatiza o reconhecimento de sua importância.

### Limitações do estudo

Este estudo apresenta algumas limitações que devem ser levadas em consideração. Em primeiro lugar, é importante ressaltar que pesquisas relevantes podem não ter sido publicadas em periódicos acadêmicos pesquisados, e documentos governamentais e anais de eventos científicos que poderiam conter informações pertinentes podem não ter sido incluídos na análise. Além disso, as revisões de escopo não incluem uma avaliação crítica das metodologias empregadas nos estudos incluídos, visto que diferentes instrumentos foram utilizados na mensuração dos dados. Reconhecemos que essa diversidade metodológica pode ter impactado na qualidade dos estudos apresentados nesta revisão. No entanto, ressaltamos que mais pesquisas são necessárias para uma investigação mais aprofundada sobre essa síndrome e sua relação com o trabalho dos policiais.

### Contribuições para a área da Segurança Pública, Saúde do Trabalhador, Saúde Mental e Saúde Pública

Este artigo se propõe a subsidiar a compreensão dos fatores que contribuem para o *burnout*, tendo um impacto significativo nas áreas da Segurança Pública, Saúde do Trabalhador, Saúde Mental e Saúde Pública. Sua contribuição para a produção de conhecimento sobre essa temática é notável, avançando o entendimento e proporcionando insights para futuras pesquisas e políticas direcionadas a essa população. Além de fornecer uma base sólida para estudos subsequentes, este artigo sugere a necessidade de medidas e políticas voltadas especificamente para os policiais, com o intuito de promover a saúde física e mental desses profissionais. Ações intervencionistas são essenciais para garantir o bem-estar dos policiais e prevenir o surgimento do *burnout*. É fundamental que sejam desenvolvidos projetos que visem melhorar a qualidade de vida no trabalho dos policiais, bem como incentivar pesquisas científicas para avaliar os resultados e impactos dessas iniciativas em diferentes contextos regionais. Portanto, estratégias e políticas públicas direcionadas à prevenção do *burnout* entre os policiais são urgentemente necessárias e devem ser implementadas.

## CONCLUSÕES

Houve predominância de pesquisas realizadas no Brasil e nos Estados Unidos, a maioria envolvendo profissionais do sexo masculino. Foram identificadas características laborais que favorecem o surgimento da SB entre o público pesquisado, assim como fatores que influenciam sua ocorrência. Apesar de se observar que há países com melhores condições de trabalho para os policiais em termos de segurança, constatou-se que mesmo assim eles são afetados por essa síndrome.

Foram identificados diversos fatores que contribuem para o surgimento da SB, incluindo características individuais e laborais, sendo alguns específicos dos policiais quando comparados a outras profissões. Os resultados dos estudos selecionados nesta revisão revelaram a complexidade desse fenômeno, destacando a importância de continuar discutindo os aspectos laborais desse público no campo da Saúde Mental e da Saúde do Trabalhador, tanto por gestores públicos quanto por entidades de classe.
